# Understanding Needs, Breaking Down Barriers: Examining Mental Health Challenges and Well-Being of Correctional Staff in Ontario, Canada

**DOI:** 10.3389/fpsyg.2020.01036

**Published:** 2020-07-10

**Authors:** Rosemary Ricciardelli, R. N. Carleton, James Gacek, Dianne L. Groll

**Affiliations:** ^1^Department of Sociology, Memorial University of Newfoundland, St. John’s, NL, Canada; ^2^Department of Psychology, University of Regina, Regina, SK, Canada; ^3^Department of Justice Studies, University of Regina, Regina, SK, Canada; ^4^Psychiatry, Queen’s University, Kingston, ON, Canada

**Keywords:** correctional work, well-being, fixed term/casual labor, recognition, occupational stress injuries, public safety personnel

## Abstract

Mental health challenges appear to be extremely prolific and challenging for correctional service employees, affecting persons working in community, institutional, and administrative correctional services. Focusing specifically on correctional workers employed by the Ontario Ministry of the Solicitor General, we shed light on their interpretations of the complexities of their occupational work and of how their work affects staff. Using a qualitative thematic approach to data analyses, we show that participants (*n* = 67) encounter barriers to treatment seeking, which they describe as tremendous, starting with benefits, wages, and shift work. We let the voices of staff elucidate what is needed to create a healthier correctional workforce. Recommendations include more training opportunities and programs; quarterly, semiannual, or annual appointments with a mental health professional who can assess changes in the mental health status of employees; offsite assessments to ensure confidentiality; and team building opportunities to reduce interpersonal conflict at work and increase moral by improving the work environment.

## Introduction

Employees in correctional services have versatile occupational roles that range from direct contact with prisoners or probationers to indirect decision-making and administrative processes that oversee prisoner, probationer, and staff experiences. Across occupational positions, researchers have demonstrated the negative physical and mental health impacts of workplace violence (Baines et al., 2011); bullying and harassment^1^ (Mayhew and Chappell, 2007); work-related stress (Bourbonnais et al., 2007; Mayhew and Chappell, 2007); and non-standard (e.g., shift work) and precarious employment relations (Triplett et al., 1996; Schaufeli and Peeters, 2000). Taken together, such similar experiences can and do affect the mental health and well-being of correctional staff. For example, the few extant studies outside of Canada indicate high rates of mental disorders among staff, particularly correctional officers (COs) (Tartaglini and Safran, 1997; Lambert et al., 2010a, b; Austin-Ketch et al., 2012).

In Canada, Carleton et al. conducted a prevalence study, from September 2016 to January 2017, of mental disorders among Canadian public safety personnel, including correctional workers (Carleton et al., 2018a, b; Ricciardelli et al., 2018a). The researchers found evidence that 54.6% of correctional workers, compared to the study average of 44.5% of all PSP, screened positive for one or more mental disorders. In addition, self-reported positive screens were much higher than the diagnosed general population epidemiological rate of 10.1% for any mental disorder (Statistics Canada, 2012). Many correctional staff self-reported symptoms yielding positive screens for post-traumatic stress disorder (29%), major depressive disorder (31%), and generalized anxiety disorder (24%) (Carleton et al., 2018a, b).

Ricciardelli et al. (2019) further broke down the rates of positive screens for mental disorders among the employees of the Canadian federal correctional service, Correctional Services Canada (CSC), evidencing that nearly 30% of the CSC employees working in operational roles within prisons (e.g., COs) screened positive for post-traumatic stress disorder and 57% for any mental disorder. Many persons working in operational roles in the community (e.g., parole officers) screened positive for post-traumatic stress disorder (26%) and/or any mental disorder (45%). Among persons working in administrative roles in regional or national headquarters, 37% screened positive for post-traumatic stress disorder and 56% for any mental disorder. Accordingly, mental health challenges are evidenced among correctional service employees, affecting persons working in community, institutional, and administrative correctional services, and warrant immediate attention (Ricciardelli et al., 2018c, 2019; Ricciardelli, 2019). Moreover, to fully understand the mental health and well-being of correctional staff, the voices of correctional staff should be central to any analyses or interpretations of their experiences and in any mental health strategies developed for the future.

In the current study, we focus specifically on giving voice to correctional staff employed by the Ontario Ministry of the Solicitor General. We look specifically at employees in a provincial correctional service to shed light on their interpretations of the complexities of their occupational work and of how their work affects staff. We show that participants encounter barriers to treatment seeking described as tremendous, and starting with benefits, wages, and shift work. We let the voices of staff who, without being directly asked, volunteered their views of what is needed to create a healthier correctional workforce lead the data analysis.

### Correctional Staff Well-Being

Correctional workers may work in the community or in administrative spaces, but many work within prisons. The institutional prison work environment is often harsh and overcrowded (Public Services Foundation of Canada, 2015), laced with staffing challenges (Martin et al., 2012), and the potentiality for violence (Ricciardelli and Gazso, 2013; Ricciardelli et al., 2018c; Ricciardelli, 2019). In Canada, prison work can occur in physically poor conditions that compromise the well-being of both prisoners’ and staff’s (Poirier et al., 2008; Zarate, 2010; Sapers, 2013). Such compromises are significant given that, in the United States, Bierie (2012b) connected the physical conditions of prisons (e.g., noise, dilapidation, lack of privacy) to physical (e.g., increased headaches, back pain, and stomach aches) and mental (e.g., depression, concentration problems) health challenges for staff. Martin et al. (2012) found that staff in crowded prison facilities experience higher levels of stress due to safety concerns, increased violence, and impaired job performance. Staff also attributed chronic health issues, such as obesity, headaches, diabetes, alcoholism, and weak immune systems, to the work environment. Overall, regional and (inter)national correctional work environments have been associated with work-related stress and employee absenteeism, perceived or actual instances of victimization, burnout^2^, and mental disorders or compromised health (Lambert et al., 2009; Lambert and Paoline, 2010; Boyd, 2011; Boudoukha et al., 2013; Doucette, 2014; Carleton et al., 2018a; Harizanova and Stoyanova, 2019; Ricciardelli et al., 2019; Useche et al., 2019; Haynes et al., 2020).

In addition, prison work presents unique conditions facilitating exposures to potentially psychologically traumatic events (Canadian Institute for Public Safety Research and Treatment [CIPSRT], 2019). Researchers studying fear of victimization among correctional staff find a positive relationship between said fear and perceived job stress (Cullen et al., 1985; Armstrong and Griffin, 2004; Paoline et al., 2015) as well as burnout (Savicki et al., 2003). For example, Arnold (2017), looking at the effects of prison on prison staff, contends that, “concerns for personal safety are a very real and salient component of the job that is a statistically significant correlate of stress” (p. 290). In provincial prisons in Quebec, Canada, researchers characterized correctional work as embedded with job strain (defined as doing work that is psychologically demanding without much autonomy) and workplace harassment, leading staff to report experiences of psychological distress (Bourbonnais et al., 2007). Stress, defined as “the physiological or psychological response to internal or external stressors” (American Psychological Association, 2020), can also be produced by colleagues in correctional work, in part through psychological harassment and intimidation (Bourbonnais et al., 2007).

Supervision and relationship quality between supervisors and staff too have been positively correlated with job stress (Lambert et al., 2009; Lambert and Hogan, 2018). In France, for example, prison staff who were victims of prisoner perpetrated violence and who had also experienced high levels of emotional exhaustion, depersonalization, stress, intrusion, avoidance, and hyper-reactivity were evidenced at increased risk for developing post-traumatic stress disorder (Boudoukha et al., 2013). Overall, correctional work is demonstrably challenging, resulting in stress and compromised well-being; however, researchers have rarely focused on treatment-seeking patterns of correctional staff or perceived barriers to correctional staff accessing support or treatment.

In a study on workplace barriers to treatment seeking among public safety personnel (e.g., communications officials, firefighters, paramedics, police) that included correctional service employees, Ricciardelli et al. (2018b) found evidence that systematic and individual factors may discourage PSP from seeking care. PSP reported factors, including stigma (i.e., being seen seeking help for mental health), understaffing, and perceived occupational repercussions (e.g., not being promoted if one seeks treatment for a mental disorder), as major barriers to treatment-seeking (Ricciardelli et al., 2018b). Relatedly, police are also unlikely to seek treatment for mental disorders, even when their organization makes mental health support the norm rather the exception (Wester et al., 2010; Reavley et al., 2018).

Overall, a body of literature exists that look at prisoner experiences of mental health care, including from the perspective of prisoners (see Drapalski et al., 2009; Mitchell and Latchford, 2010; Shaw and Morgan, 2011); however, to our knowledge, scholars have yet to study the barriers to care and treatment seeking experienced explicitly by correctional staff, despite the broadly accepted need for treatment (Oliphant, 2016).

## Current Study

Correctional work involves a versatile assortment of occupational roles with a multitude of responsibilities toward society, staff, prisoners, and self. Such work can affect all those working in correctional services, beyond those who appear to work in direct and frequent contact with prisoners. Correctional services workers employed in institutional, community, or administrative roles share experiences of either direct (e.g., witnessing, intervening in incidents) or indirect (e.g., report reading) exposures to potentially psychologically traumatic events (Perez et al., 2010; Bierie, 2012a; Carleton et al., 2019; Ricciardelli and Power, 2020; Ricciardelli et al., 2020). Despite variation in the degree of exposure, experiencing potentially psychologically traumatic events in whatever form appears associated with an increased risk of screening positive for a mental disorder (Carleton et al., 2018a, b; Ricciardelli et al., 2018a, b). In the current study, we aimed to recognize and respond to the mental health needs of correctional staff as put forth by employees working for the Ontario Ministry of the Solicitor General, specifically, correctional staff. We also unpack perceived treatment-seeking barriers that affect the well-being of staff and shed light on what staff perceive as challenges to working for correctional services in Ontario.

## Materials and Methods

The current article data emerged as part of *the mental health and well-being study*, designed to begin to assess the prevalence of mental health challenges and mental disorders among correctional staff working in all areas of correctional employment (e.g., institutional, community, and administrative corrections) (for more information about the larger study, see Carleton et al., 2020). Employees of correctional services in Ontario were asked to complete an anonymous and confidential online survey from December 8, 2017 to June 30, 2018. The survey followed established guidelines for web-based surveys (Ashbaugh et al., 2010). Questions included demographics and self-report assessments of various symptoms, as well as open-ended response items. For the analysis in the current study, the data were taken from the survey item, “If you have any additional information you would like to provide or additional feedback, please feel free to do so below.” The particular item did not solicit any specific comments about a specific topic; instead, the item was intentionally vague to create a space for participants to comment on whatever was on their mind while completing the survey. Thus, the data are particularly rich and meaningful, addressing the more significant factors impacting correctional service workers tied to their feeling about the effects of their employment on self, their needs, and the challenges faced in their occupation around seeking support and feeling valued as correctional employees.

Correctional service workers were recruited for participation through their government-issued email by communications put forth by the employer (the Ministry of the Solicitor General) and the Ontario Public Service Employees Union (OPSEU) informing about the survey and how to participate. Specifically, the employer sent a memo about the study and a joint employer–union memo was also emailed about the study to invite participation. Participants were also informed about the study when the employer presented on different topics to front line staff. Participation was voluntary and the email circulated provided a link to route the potential participant to the start of the survey. At the start of the survey, each participant was provided with a randomly generated unique access code, which allowed participants to log in to their anonymous survey responses from any computer. As such, participants could exit and return to continue the survey without having to start anew. Depending on the scope of the answers provided in open-ended items and reading speeds, among other factors, the survey required between 25 and 40 min to complete.

Over 1,300 individuals provided informed consent and accessed the survey with complete (or near complete) data; however, only 67 provided an answer to the open-ended “final comment” item (see Table 1). The data from the 67 participants were used for the current study.

**TABLE 1:** Demographics of all participants who responded to the item: “If you have any additional information you would like to provide or additional feedback, please feel free to do so below.”

**Variable**	**% (n)**
**Sex**	
Male	53.7 (36)
Female	46.3 (31)
**Age**	
19–29	11.9 (8)
30–39	22.4 (15)
40–49	34.3 (23)
50–59	22.4 (15)
60 and older	9.0 (6)
**Marital status**	
Married/common-law	71.6 (48)
Single	13.4 (9)
Separated/divorced/widowed	13.4 (9)
Remarried	1.5 (1)
**Occupation**	
Corrections officer	82.1 (55)
Probation/parole officer	17.9 (12)
**Education**	
Graduated high school	9.0 (6)
Some post-secondary (less than 4 year college/university program)	40.3 (27)
University degree/4 year college or higher	46.3 (31)
Other	4.5 (3)
**Years of service**	
More than 15 years	46.3 (31)
10–15 years	13.4 (9)
4–9 years	11.9 (8)
Less than 4 years	20.9 (14)
Missing	7.5 (5)

### Strategy of Analysis

The data were analyzed using a thematic inductive process (Hesse-Biber and Leavy, 2004), specifically a constructed semigrounded emergent theme approach (Glaser and Strauss, 1967; Ricciardelli et al., 2010; Charmaz, 2014) that involved a multistep coding process utilizing QRS NVivo Pro. First, we imported the participant dataset into NVivo using the autocode function to ensure that each participant was autocoded as a unique case and their data tied to their attributes (i.e., demographic and other such factors; ensuring that the data are labeled such that, for example, gender and occupation are able to be controlled for during analyses). The autocode function enables each respondent’s data to be classified into “parent” (e.g., primary themes) and “child” (e.g., secondary themes) nodes, which allows for the identification of primary and secondary themes within each topic identified. In other words, all responses that were thematically similar (i.e., participants voiced similar concerns) were grouped under the topic of note, which was then coded into primary themes and then recoded into secondary and/or tertiary themes. Independent graduate level research assistants completed multiple reviews of the imported data as they coded the dataset – gaining a sense of the data as a whole and key themes across responses (Corbin and Strauss, 2015). Axial coding was employed to ensure that only thematically relevant data were included in the current analysis (Strauss and Corbin, 1990), and an exhaustive but not mutually exclusive coding scheme was created through the repeat coding of the data. Research ethics boards at Queens University (file #6024787), University of Regina (file #2017-098), and Memorial University of Newfoundland (file #20201330-EX) approved the data collection for the current study. All quotes are used with permission in the results section, and some are edited for spelling, grammar, or readability without affecting the vernacular or tone.

## Results

All areas of correctional work may impact the mental health and well-being of staff (Carleton et al., 2018a; Ricciardelli et al., 2019); however, respondents for the current study voiced feelings that the impact of correctional work on mental health remains under-recognized. For instance, participant 89, a male correctional officer, explained that: “working as a CO for Ontario Corrections has been completely unsatisfying and overall detrimental to my personal life”, thus explaining that the effects of their work has negatively affected both their self and their personal living. Other respondents reported:

Corrections are a very mentally and physically demanding career that is very difficult to understand. Being keepers of people has demands that should not be misunderstood. This career is a meat grinder (Participant 162, correctional officer, male).

I have worked as a rehab officer… I have suffered being harassed, bullied, and discriminated against in my workplace. Sometimes I have felt supported and other times it has been almost impossible to walk in the building. I am still having residual issues as a result of the abuse (participant 357, female, rehabilitation officer).

…Smaller incidents that are repeated many times over a year or even an entire career can also have a detrimental impact on one’s stress level at work and overall mental health… I believe that these incidents [having urine thrown at them] should they happen to a person on a regular basis could have a significant negative impact on ones mental health, and I say that from the experience of having this happen to me. I believe that things like this in this line of work are more likely to be “swept under the rug,” and belittled for what their true value is (participant 360, female, correctional officer).

Each of the three participants reported different aspects of working in correctional services that can negatively affect their well-being but also demonstrate, at least in part, the scope of challenges in which they must navigate. Participant 162, called the job a “meat grinder” speaking to the complexities of working with incarcerated persons for those in institutional corrections. Participant 357 reported interpersonal challenges that must also be navigated at work, for those working in all areas of correctional services. Participant 360 reported on the often overlooked impact of an accumulation of smaller incidents on well-being. Together, the participants highlight how working in correctional services can negatively impact mental health and well-being. We structure the following results first by addressing the need for mental health to be recognized by the employer and second by identifying factors that make staff feel underappreciated in correctional work. We also shed light on the treatment-seeking barriers related to mental health (i.e., feelings that their wages do not compensate for the scope of their work, the effects of forced overtime or casual employment, inadequate benefits).

### The Need to Recognize Mental Health

Despite increasing recognition of the mental health challenges faced by PSP, including those in correctional services (Oliphant, 2016), participants put forth, without direct questioning, that the employer and at times themselves need to more fully recognize staff mental health needs and the complexities of their work. Participant 194, a female probation officer noted that “the employer needs to address the epidemic of mental health in the workplace…”, thus suggesting that the problems of compromised staff mental health in correctional services requires more understanding and attention. Another respondent, a male correctional officer, participant 299 reported: “The Ministry has to start again being more concerned with their staff that uphold their facilities and maintain the stronghold of incarcerating offenders. Without your front line staff, you have nothing.” Thus, across correctional occupations, there were reports that more needs to be done to support mental health and well-being. As previously noted, correctional work impacts staff, to varying degrees, who too often keenly feel underrespected. Participant 119, a self-identifying female working in prison, explained that:

I am fortunate to have left the front line but hope that governments and communities will begin to recognize the importance and seriousness of the work that is done by those workers that perform the duties that most people would/could not do that may, at times, require understanding, time for self-care, help from others, and respect for the important work that they do to protect us all.

The participant’s words reveal she feels undervalued for the work she performs on the “front line”, but also that the “seriousness” of the work is under-recognized. Such feelings were exacerbated in cases where staff felt less supported with fewer rights than held by those in custody or under supervision. Participant 254, a male correctional officer, reported: “Correctional Staff should be expected to have equal rights and access to mental health services as offenders/inmates/clients. It should be expected that Correctional Staff have the same and equal rights when it comes to dealing with verbal, physical, emotional, and mental abuse that is afforded to and expected by offenders/inmates/clients”. The notion that staff feel they have fewer rights than those under their care is troubling and can reasonably be expected to act as an exacerbating factor impacting staff well-being.

Participants also reported the need for increased awareness among staff about their own state of mental health. Some respondents, like Participant 302, male, correctional officer, reported gaining recognition that they “may have some mental health or stress related issues related to work”. Participants reported repeated exposure to potentially psychologically traumatic events as impacting their own mental health and observing changes in other staff as well. Other participants reflected on their personal experiences and state of mental health, reporting inadequate supports and time for the necessary “self-care” to cope with their occupational realities. Participant 205, a male correctional officer, for example, explained:

…wellness activities should be encouraged and supported. Examples would be work out/gym time, yoga, meditation, mindfulness courses, and nutrition classes. Perhaps spiritual support for those inclined. I feel this type of investment in the health of employees would pay dividends in reduced sick time, higher engagement and job satisfaction. I work in a facility that has an ability to feed 1,200 inmates but offers only junk food vending machines to employees.

Respondents voiced a need for their employer to recognize the mental health and well-being needs of staff; nevertheless, some staff were disillusioned and felt the employer was unwilling or unable to impact change, like participant 777, a female probation officer, who reported:

I believe that the employer and the system, in general, can do things to improve the situation, but they are either unwilling or unable (restrictive policies or lack of funding), which is resulting in a loss of the good people.

Even more participants expressed hope that positive changes would be forthcoming from the employer in the future. The overall sentiment–held by a majority–was hopefulness toward improvements in staff wellbeing. For example, Participant 1054, a male correctional officer, reported the sentiment and associated concerns:

The issue herein lies with the management teams and the National and Provincial Governments to come to the realization that officers in corrections need support and treatment, debriefing, and programs to help them outside of the workplace so that they come to work productively and without malice. Programs are needed for early detection of problems; peer programs donams are needed for early detection of problems come to work productively and withzation that officers in corrections need support and treatment, alth of employees woulde you to help the Ministry of Corrections to see officers as humans, not as numbers.

Beyond expressions of hope, participants also put forth recommendations that the employer should provide more training opportunities and programs [e.g., “I think staff should get mental health first aid” (participant 284, female, recreation officer)]; quarterly, semiannual, or annual appointments with a mental health professional who can assess changes in the mental health status of employees (e.g., “Mental health assessment on an annual basis”; participant 402, male, CO); offsite assessments to ensure confidentiality [e.g., “…An hour with a counselor, while wearing civilian clothes, offsite where they can read the paper if they want, or actually discuss what they’ve seen or experienced so far” (participant 765, male, CO)]; and team-building opportunities to reduce interpersonal conflict at work and increase moral by improving the work environment (e.g., “team building, everyone has to give everyone a chance and be open and accepting. It’s vital that all staff members respect each other…”; participant 484, male, CO). In addition, participants reported central concerns as impeding their self-care, their feeling of being appreciated and valued, and their treatment-seeking mental health.

#### Fixed Term Employment

For institutional correctional services in Ontario, Canada, fixed-term (also referred to as casual) employment refers to employees who receive a percentage of pay in lieu of insured benefits upon completion of a month of continuous service. By definition, said employees are not permanent in their position (i.e., they do not have a “home” position to which they are tied; personal communication, Flood to Ricciardelli, 2020, Jan. 20)^3^. Fixed-term employees lack long-term job security, which is personally challenging, and also face challenges in the workplace as they are without seniority or regularity at work, and accordingly, the occupational positioning (e.g., being a fulltime scheduled employee) that is awarded a consistent degree of respect from fellow staff. The reliance on casual employees, particularly given the existence of fulltime scheduled shift employees, creates a hierarchy among correctional staff working in institutions. In consequence, workplace moral is impacted, as is the intensified vulnerability experienced by fixed-term staff. As participant 254, a male CO, reported:

Improvements can be made to workplace morale and be made better overall by making all Correctional Officers full time or scheduled regular shifts in advance. This would improve dealing with the daily stressors of the regular duties Correctional Officers face. Irregular shifts compound the stress and problems faced on duty by FIXED TERM Correctional Officers.

Focused on the impacts of fixed-term employment on moral and fixed-term staff, the words of participant 254 exemplify the vulnerability that comes with said employment within a penal institution or correctional setting. Such employment causes strains to both the fixed-term and fulltime scheduled staff, the former because they never know when they will be at work, and the latter because of the lack of regularity and associated safety tied to having a steady partner and experienced staff working in select positions and postings. Evidenced in the quote is that staff morale is compromised by the uncertainty, for all staff, tied to not knowing who they will be working alongside. As such, the causal employee is not entering a welcoming staff work environment given they are not scheduled and constitute a “surprise”.

Such organizational stresses can impact staff well-being (Ricciardelli, 2018; Carleton et al., 2020). Participant 289, a casual or fixed-term male CO, explained some challenges with working in a casual position:

Fixed-term employees experience far greater levels of stress than fulltime as we do not [know] where we will be working, what our post will be, and therefore must know every post in the building and the appropriate policies all without vacation time and health benefits.

As evidenced in the words from participant 289, stress tied to fixed-term employment is inherent to the occupational position. For example, not having benefits yet working in a high-risk occupation with the potentiality for exposure to physical violence is inherently concerning. In addition, the lack of vacation (or downtime) impedes time to engage in self-care for said employees, exacerbating stress or susceptibility to occupational stressors (e.g., difficulties with management, responding to altercations). Nonetheless, participants reported casual employment as a commonly used form of necessary employment for staffing facilities.

There was also a shared concern across participants about “being called into work” (Participant 462, female, correctional officer). Participants, fixed-term or scheduled, found either being called into work an unscheduled shift or being held back to work extra hours on a shift stressful. Such was particularly the case for fixed-term employees, like participant 462, who reported:

…shift work is hard enough let alone taking last minute phone calls for a nightshift that was not prepared for or being called into work after only 8 h of completing a nightshift. It would be extremely beneficial if casual employees could put in for preferred work hours regarding nights or days. Of course this type of job wouldn’t guarantee it 100%, but it might ease some of the burden of shift work for casual employees rather than bouncing back and forth, often numerous times in a week between day shifts and night shifts.

The challenge for fixed term employees involves the unpredictability of their work and the irregularity of their shifts [e.g., “bouncing back and forth, often numerous times in a week between day shifts and night shifts” (participant 462)], which impacts their sleep hygiene, and their life both inside and outside of work. Fixed-term employees are broadly impacted by irregular and unscheduled shifts and lack of benefits, which serve as barriers to treatment-seeking. The former makes attending and scheduling appointments with medical professionals challenging for fixed-term staff, while the latter financially bars engagement in treatment or preventative measures tied to mental health.

#### Wages and Benefits

Participants voiced dissatisfaction with their current wages and mental health benefits, specifically feeling that their “current pay does not reflect the emotional, physical, and verbal abuse correctional officers receive daily. Additionally, the amount of time spent away from our families to expose ourselves to the correctional environment is unreasonable” (Participant 279, male, CO). The lack of benefits, genuinely non-existent for fixed-term employees, was a voiced concern as was the scope of benefits fulltime scheduled employees are entitled to. Staff require benefits to support seeking mental health treatment, but participants felt that their benefits were inadequate and served as a barrier to treatment-seeking. For example, participant 580, female, probation officer, explained:

Although I have and would seek out treatment for mental health, our current benefits plan is an obstacle to employees receiving adequate mental health treatment (e.g., only $50 per session is paid for [a] psychologist). This is cost-prohibitive for me and most employees. Also, one has to seek treatment on [their] own time with little accommodation from employer even when [the] “injury” is a workplace one. This can be an additional stressor. Employees should have access to fully paid mental health treatment especially if due to work-related exposure.

As evidenced in participant 580’s words, a dyad of factors culminate in seemingly unsurpassable barriers for treatment-seeking: benefits fail to cover costs of treatment and a lack of employer accommodation for treatment-seeking. Participants voiced valuing mental health support provided by peers who are not under the employ of the Ministry because of concerns about confidentiality. Particularly after critical incidents (e.g., potentially traumatic events that are possibly life threatening or compromising to staff well-being) at work do participants feel intervention or debriefing was particularly necessary. For example, participant 553, a female nurse, explained that:

I feel when there are critical incidents that require support for staff this should be provided by an outside agency of trained professionals…in a timely confidential manner. Currently CISM is a team of peer volunteer supports who are called on. Many staff identify this as a concern as they are peers.

Participant 553 describes preferring treatment at the hands of professionals rather than peers and valuing treatment being provided quickly after such incidents and with confidentiality – a confidentiality perceived as not guaranteed to the same extent by peers. Confidentiality was described as particularly important, and perceptions of the possibility for confidentiality to be breached was an undisputed barrier that participants reported for seeking support or participating in debriefings. For participant 801, a female probation officer, living in a small town, where mental health services were in short supply, was another barrier to treatment for mental health:

I have and would go out of area for any medical treatment but had to go on my own time of fear anyone would find out so have had to use vacation days for counseling and medical. Also in small towns you know all the service providers from work so it doesn’t feel confidential and would be awkward/embarrassing to use local mental health services.

The words of participant 801 are particularly relevant given that community and institutional correctional services are located all over the province and thus many under the employ are living in small or rural more remote areas.

Others, such as participant 141, a female probation officer, faced further barriers; specifically, “I will always help others, without judgment… but would never seek help myself (so basically am fucked).” This participant, like others, inadvertently describes a stigma attributed to mental health or perceived as tied to persons with mental health concerns. Others also noted stigma as a barrier to treatment seeking:

[There is] still a large stigma over mental health issues in corrections. Once PTSD was acknowledged some of those we know to be “abusers” of sick time went off, it’s a running joke. Unfortunately, I still have my mentality from starting in 1996 in corrections, you never talk about your problems and you trust no one (participant 398, female, correctional officer).

Some staff are expected to work such long hours that having healthy periods off just can’t happen without falling behind at work and adding more stress. It can be a tough place to work with limited supports and it still has the stigma that you have to be tough to work in a jail and are expected to be okay after all events and to not show emotion or break down (participant 780, female, health care).

…people who are managers see any mental health issues as a sign of weakness and target you so as to make you quit or they can get you on constructive dismissal. I have no problems talking about my PTSD if it can help even just one person (participant 1036, male, correctional officer).

Participant 398 reported their attitude is “old school” and attributable to having started working in correctional services in 1996 when mental health and disclosing having a mental health challenge were taboo. Participant 398 showcases the stigma tied to persons who “went off” on sick leave for post-traumatic stress disorder. Here, the credibility of their colleague’s diagnosis is doubted–which in essence signifies a stigma is imparted on persons who claim post-traumatic stress disorder and take leave as a result. Participant 780 described a culture surrounding the need to self-present as “tough” and “not show emotion or break down” within correctional services. Further, participant 1036 demonstrated the changes in stigma by noting that, although having a mental illness may still be viewed as a sign of “weakness” and may even encourage discrimination, there are people still willing to talk about their own mental health challenges. The nuances around mental health stigma may have changed over the years, taking new forms, while remaining pervasive in impacting perceptions, interpretations, attitudes, and behaviors.

Overall, our participants echo others in reporting a multitude of barriers to treatment seeking. Participants continue to report believing that mental health is the responsibility, even obligation, of the employer, who needs to provide support through their benefits packages as a result of their occupational realities. Participant 453, male, management, reported:

Front line staff need covered benefits to assist in mental health issues. Accumulation of incidents that are not normal working conditions (death threats, urine and feces being thrown at you, physically assaulted, threats to your family, suicides, etc.), have a prolonged effect on staff. There needs to be help from professionals provided for staff to keep them functional and working instead of burning out and crashing then becoming a liability.

## Discussion and Implications

The nuances regarding mental health may shift between correctional staff; nevertheless, our participants recognize the need to progress further talks about mental health in the carceral workplace. Respondents indicate that both correctional staff and the employer need to have more recognition of staff mental health needs, particularly in the context of the occupational nuances of the job. Especially when one takes into consideration how dynamic relationships between supervisors and staff in correctional settings inform whether job-related stress is reduced (Lambert et al., 2009; Lambert and Hogan, 2018), our respondents mirror similar concerns toward the respect (or perceived lack thereof) they receive while they are working. Other participants expressed a need for further awareness of their own personal state of mental health, while at the same time reporting perceiving inadequate access to support and time for self-care.

Prior researchers have underscored the negative impact of workplace stressors on staff (Bourbonnais et al., 2007), including bullying and harassment (Mayhew and Chappell, 2007). Respondents offered recommendations, such as employees receiving mental health first aid, and consistently offered regular appointments with mental health professionals that occur offsite to safeguard confidentiality, toward responding to mental health challenges. There were also arguably more easily implemented suggestions such as team-building exercises that may assist in reducing interpersonal conflict between coworkers. Such efforts may support correctional staff in feeling appreciated and valued, reduce workplace stress via interpersonal demands (see Mayhew and Chappell, 2007), and may help improve treatment-seeking as needed.

Prior researchers have focused on correctional workers’ job tenure and the relationship between tenure and turnover intent; specifically, as job tenure increases, the desire to leave or quit correctional services decreases (Lambert and Paoline, 2010; see also Lambert et al., 2010b). To our knowledge, no one has directed attention toward precarious employment situations among correctional service workers, particularly fixed-term vs. fulltime employment. Respondents posited that hierarchies among correctional staff working in institutions are created between fixed-term and fulltime employees, and “workplace morale” suffers in the process. Furthermore, the lack of vacation (or downtime) for fixed-term employees impedes their ability to engage in self-care, or to take time away for health services, which may exacerbate existing stress or increase susceptibility to new stressors.

Participants reported concerns about their rate of pay as well as benefit packages, specifically their benefits being inadequate and creating barriers to mental health treatment-seeking. In addition, respondents raised concerns about confidentiality; specifically, the possibility for (or fear of) confidentiality to be breached appears to act as a barrier to support seeking. Such concerns, as well as doubting the credibility of a colleague’s compromised well-being, may reinforce that efforts to seek mental health care remain impeded by the stigma tied to treatment-seeking for mental health (Oliphant, 2016; Ricciardelli et al., 2018b). Confidentiality, stigma, and credibility were intricately connected in participants’ responses, highlighting how the nuances surrounding employee mental health stigma remain an ongoing concern.

As evidenced, recognition appears central for assisting correctional staff in their efforts to lessen the stigma tied to treatment-seeking or mental health in correctional workplaces. Consistent with Oliphant (2016), recognition remains an important first step to better assist correctional staff in their efforts to lessen stigmatization of mental health issues in their correctional workplaces. There is no doubt that correctional work continues to be challenging; mental health issues and barriers to treatment and support should not be additional challenges. In the current study, we begin to make inroads toward describing and informing how a small sample of correctional staff employed in Ontario experience barriers to care and treatment-seeking experienced explicitly in their work. Referring to public safety personnel in general, Carleton et al. (2019, p. 50) underscore the importance of “adequate, pervasive, and evidence-based mental health care treatments and supports to mitigate the negative impact of repeated exposures to diverse potentially traumatic event exposures to public safety personnel.” Echoing a similar sentiment, if we are to effectively break down barriers to mental health challenges for staff in Ontario’s correctional services, a sustained dedication toward mental health support for correctional workers must become clearly, consistently, and comprehensively established.

### Limitations and Caveats

We designed the survey in collaboration with the Ontario Ministry of the Solicitor General (e.g., determining scales and questions) and focused on the specific interests, needs, and concerns of Ontario correctional employees. The intention was to develop a survey that assessed the scope of the mental health needs and can inform the creation of processes for identification of, proactive engagement with, and treatment for correctional employees. Like all qualitative results, caution is necessary when considering generalizability. In addition, our study is limited by the small sample size, which is quite modest given that the sample includes only employees who provided a comment on an open-ended question at the end of the study. Moreover, we did not ask participants any questions related to the emergent themes; instead, the themes arose from the open-ended responses participants provided to a request for final comments.

## Conclusion and Future Research Needs

Mental health needs and the associated barriers to support for correctional service employees remain an ongoing challenge, despite significant gains in recognizing and respecting the mental health challenges and barriers to treatment-seeking raised by participants. We must consider also the practical intervention strategies that could be implemented to tackle the main threats to correctional workers’ well-being. For example, strategies designed to enhance the perception of managerial support and organizational justice should be further developed in correctional settings, as these strategies may significantly prevent the occurrence of counterproductive work behavior associated with employees’ mental health symptoms, such as presenteeism and workplace bullying (Guglielmi et al., 2018; Mazzetti et al., 2019). Additionally, respondents’ concerns included insufficient benefits and wages, a need to recognize mental health, and how the interacting challenges impact mental health treatment-seeking, all of which provide avenues for future research and policy development. For example, the effects of casual employment on “workplace morale” are unclear and likely represent a needed area of future investigation. Furthermore, despite the wide acceptance that mental health challenges are significant concerns, there are very limited published data regarding mental disorder rates among Canadian correctional employees, and even less focused on Ontario correctional services. The absence of such data problematically limits quantifying the scope of the challenges facing correctional workers. Without an understanding of the scope, petitioning for resources to support research or treatment for staff is compromised. Finally, with no data on the current state, there is no way to assess whether efforts to help manage mental disorders are making a difference for correctional employees or about how to improve such efforts. Considering the current study results, addressing the diverse occupational needs of the correctional workforce appears to be a potentially critical component of an effective mental health strategy.

## Data Availability Statement

The datasets presented in this article are not readily available because of anonymity and confidentiality requirements. Requests to access the datasets should be directed to the corresponding author.

## Ethics Statement

The studies involving human participants were reviewed and approved by the Research Ethics Boards at Queens University (file #6024787), University of Regina (file #2017-098), and Memorial University of Newfoundland (file #20201330-EX) approved the data collection for the current study. The patients/participants provided their written informed consent to participate in this study. Written, informed consent was obtained from the individual(s) and/or minor(s)’ legal guardian/next of kin for the publication of any potentially identifiable images or data included in this manuscript.

## Author Contributions

RR was involved in all aspects of the manuscript. RC did a final review and edit of the manuscript. JG drafted the discussion. DG did the table. All authors contributed to the manuscript.

## Conflict of Interest

The authors declare that the research was conducted in the absence of any commercial or financial relationships that could be construed as a potential conflict of interest.

## Footnotes

1 While the terms “bullying” and “harassment” are often used interchangeably in scholarship and society, in the current article, we define bullying as per Johnson et al. (2015, p. 452) who use bullying to refer to “frequent and persistent negative acts directed toward one or more persons in the workplace.” Although harassment is similar to bullying, insofar as one person hurts another through cruel, offensive, or insulting behaviors, we define harassment in the current article as “unwanted conduct that is based on race, color, religion, sex (including pregnancy), national origin, age (40 or older), disability or genetic information” (United States Equal Employment Opportunity Commission, n.d., p. para 2).

2 Burnout, as defined by Maslach and Leiter (2016, p. 103), is “a psychological syndrome emerging as a prolonged response to chronic interpersonal stressors on the job. The three key dimensions of this response are an overwhelming exhaustion, feelings of cynicism and detachment from the job, and a sense of ineffectiveness and lack of accomplishment.”

3 In contrast, regular permanent employees working in institutions have a “home position”; thus, they can be seconded to other positions but always have a “home” position to return to (personal communication, Flood to Ricciardelli, 2020,Jan. 20.).
